# Altered Network Communication Following a Neuroprotective Drug Treatment

**DOI:** 10.1371/journal.pone.0054478

**Published:** 2013-01-22

**Authors:** Kathleen Vincent, Joseph S. Tauskela, Geoffrey A. Mealing, Jean-Philippe Thivierge

**Affiliations:** 1 School of Psychology, University of Ottawa, Ottawa, Ontario, Canada; 2 Institute for Biological Sciences, National Research Council, Ottawa, Ontario, Canada; Ospedale Pediatrico Bambino Gesù, Italy

## Abstract

Preconditioning is defined as a range of stimuli that allow cells to withstand subsequent anaerobic and other deleterious conditions. While cell protection under preconditioning is well established, this paper investigates the influence of neuroprotective preconditioning drugs, 4-aminopyridine and bicuculline (4-AP/bic), on synaptic communication across a broad network of *in vitro* rat cortical neurons. Using a permutation test, we evaluated cross-correlations of extracellular spiking activity across all pairs of recording electrodes on a 64-channel multielectrode array. The resulting functional connectivity maps were analyzed in terms of their graph-theoretic properties. A small-world effect was found, characterized by a functional network with high clustering coefficient and short average path length. Twenty-four hours after exposure to 4-AP/bic, small-world properties were comparable to control cultures that were not treated with the drug. Four hours following drug washout, however, the density of functional connections increased, while path length decreased and clustering coefficient increased. These alterations in functional connectivity were maintained at four days post-washout, suggesting that 4-AP/bic preconditioning leads to long-term effects on functional networks of cortical neurons. Because of their influence on communication efficiency in neuronal networks, alterations in small-world properties hold implications for information processing in brain systems. The observed relationship between density, path length, and clustering coefficient is captured by a phenomenological model where connections are added randomly within a spatially-embedded network. Taken together, results provide information regarding functional consequences of drug therapies that are overlooked in traditional viability studies and present the first investigation of functional networks under neuroprotective preconditioning.

## Introduction

Neurons represent the cellular building blocks of the brain and their complex intercellular mechanisms of communication provide a foundation for higher-order functional networks to emerge. One challenge in developing pharmacological therapies for the brain is to design experimental models that retain key features of network function. Experimental procedures based on ischemic preconditioning have uncovered effective strategies to prevent neuronal death by activation of an endogenous stress response prior to an ischemic stroke [1], [2]. One drug combination has recently been shown effective in preventing neuronal demise through preconditioning. It combines 4-aminopyridine (4-AP), a selective voltage-gated potassium channel blocker, and bicuculline (bic), a GABA_A_ receptor antagonist [3].

While previous work has shown that 4-AP/bic provides *in vitro* cell viability under combined oxygen and glucose deprivation, its effects on communication within synaptic networks have yet to be examined. This question arises in light of recent work representing functional brain networks (i.e., maps of the statistical interactions between neurons) as a highly complex system that can be understood by applying principles of graph theory [4]. Various neurophysiological impairments are accompanied by a breakdown in graph-theoretic properties [5]. Does short-term exposure to 4-AP/bic, a potential neuroprotective measure for stroke, cause long-term changes in the functional organization of cultured cortical networks?

Here, we employ multi-electrode arrays to record from networks of cultured cortical neurons. In this preparation, dissociated neurons are plated over a grid of electrodes that record extracellular field potentials. Multi-electrode arrays record neuronal activity at a spatial resolution that is a compromise between microscale (i.e., single synapses) and macroscale (on the order of millions of neurons, as in neuroimaging) approaches [6]. The spatiotemporal patterns of bursting activity revealed by multi-electrode arrays provide a glimpse of the underlying functional connectivity in the neuronal network [7], [8]. Highly non-random graph-theoretic properties have been characterized in functional networks of dissociated neurons, including a small-world organization [9], [10]. In these networks, functional interactions are dominated by weak pairwise correlations that allow neurons to communicate with few intermediate relay steps.

To characterize how ischemic preconditioning affects the small-world organization of *in vitro* cortical cultures, we reconstructed functional connectivity using a permutation test before and after 4-AP/bic exposure. We examined functional networks in terms of their graph-theoretic properties, and devised a phenomenological model that offers a link between these properties in spatially-embedded networks. Our results uncover specific aspects of functional connectivity that undergo long-term alterations as a result of preconditioning, supporting the perspective that graph theory can provide useful markers of pharmacological impacts on neuronal circuits.

## Materials and Methods

### Tissue Culture and Recording

The protocol for preparing the primary neuronal cultures was approved by the Institute for Biological Sciences Animal Care Committee (National Research Council Canada). Here, we provide an overview of methods, and refer elsewhere for further details [3].

**Figure 1:**
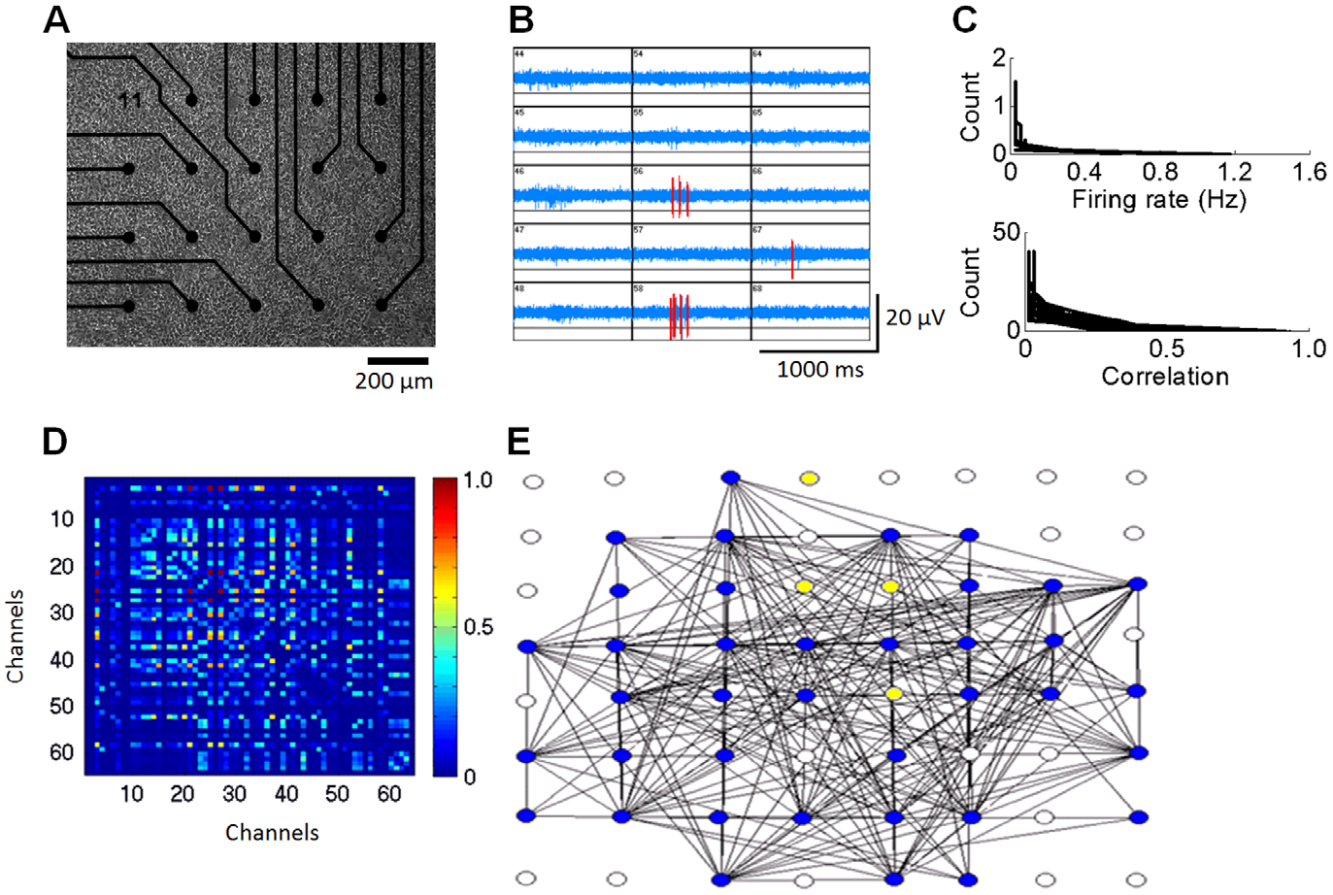
Experimental procedure for reconstructing functional networks of cultured cortical neurons. A. Rat cortical neurons plated on a 64 electrode microelectrode array (MEA) (only a subset of electrodes shown). Electrodes are spaced 200 µm apart and record the extracellular activity of nearby neurons. **B.** Example of extracellular spikes measured over a subset of 15 electrodes simultaneously. Red: spikes. Blue: background activity. **C.** Histograms of mean firing rates per channel (top) and cross-correlations between pairs of channels (bottom), taken over all DMSO cultures recorded at DIV 14 (N = 5). **D.** Representative matrix of pairwise cross-correlations obtained across all recording electrodes. The diagonal (activity at each channel compared to itself) is set to zero. **E.** Graph obtained by the permutation test (see Experimental Procedures). Circles correspond to electrodes on the MEA. Lines show above-chance cross-correlations. Blue: active electrodes; white: inactive electrodes (extracellular spike rate less than 3 S.D. below that of the population); yellow: active electrodes with no above-chance correlation to other electrodes. A–D: obtained with a DMSO control culture (DIV 14).

Cell cultures were recorded using 64 electrodes placed in an 8×8 array (ALA Scientific, Germany) (Fig. 1A). Each multi-electrode array was coated with a thin layer of poly-ethylinimine, a cationic polymer that facilitates cell adhesion to the glass plate. Dissociated primary cortical neurons were prepared from 18 day prenatal Sprague Dawley rats. A 1% solution of penicillin in streptomycin was added to the suspended cells and the mixture was filtered through a 40 µm cell strainer. 1000 µl of the 1.5 M cell solution was then added to each multi-electrode array to cover the electrode surface and was placed on a stand inside a covered petri dish containing 10 mL of water. The cultures were kept in a 37°C incubator with a 5% carbon dioxide/95% air atmosphere. To control the growth of microglia, mitotic inhibitor (20 µl of FUDR/UDR) was added to each culture at 4 days *in vitro* (DIV). A 50% media change was performed once a week with essential growth media. To maintain an osmolality range of 300 to 320 mOsmol, sterile water was added daily to each multi-electrode array. Each culture was inspected daily under microscope to ensure culture quality. Only cell cultures that exhibited a dense, homogenous monolayer of healthy neurons were kept for further manipulations.

### Pharmacology

Three experimental conditions were employed. In the *preconditioning* group, cultures were exposed to 500 µM 4-AP and 50 µM bicuculline dissolved in 0.3% dimethyl siloxane (DMSO) in culture media. In the *DMSO* group, the above solution was replaced with a 0.3% solution of DMSO. The *non-DMSO* group represents treatment-free cultures. A total of 15 cultures were employed and divided equally between the three conditions outlined. Unless otherwise stated, the “control” condition described in Results refers to the DMSO group.

Previous studies have demonstrated that cultures display frequent activity one week after plating, synchronized bursting activity at DIV 14 and achieve network maturity by DIV 21 [11], [12]. Because our measure of functional connectivity is based on synchronized spiking activity (see below), we began with a baseline recording of initial (pre-drug administration) activity at DIV 14. Cultures were exposed to 4-AP/bic at DIV 15 and a second recording was obtained 24 h post exposure (DIV 16). Drugs were removed after 48 hours of exposure (DIV 17) in accordance with the timecourse of drug exposure required for effective preconditioning [3]. A total media exchange was performed in order to remove the treatment. A third recording was obtained 4 h after drug washout (DIV 17) and a final recording 4 days later (DIV 21).

### Recordings

Recordings were performed using Multi Channel System (MCS) software for microelectrode arrays. Arrays were mounted on the recording platform and capped with a sterile vented tissue culture lid to maintain sterility. Prior to each recording, arrays were given a 20 minute incubation period on the platform to equilibrate within an incubator maintained at 37°C with 5% carbon dioxide. Each recording was carried out for 20 min duration. Recording parameters were as follows: 1100.0 amplifier gain, input voltage range of −2048 to +2048 mV, sampling frequency of 5000 Hz. Low frequency shifts in the raw signal were removed using a high-pass filter with a cut-off frequency of 200 Hz.

### Spike Detection

Extracellular spike detection was performed using MCS software, with a threshold of 3 S.D. below the mean of the filtered signal at each electrode (Fig. 1B). The resulting spike data from MCS were then converted into Matlab files for offline analysis.

#### Functional connectivity

Electrodes whose activity was outside of 3 S.D. from the mean spike rate of the population were removed from further data analysis. For each remaining electrode, spike data were downsampled by separating the data into non-overlapping bins of 10 ms, then taking the maximum value (0 or 1) within each bin. Functional connectivity was calculated as pairwise cross-correlations on downsampled spike data [13], [14]. Slight alterations in the size of the bins did not drastically alter the results of this calculation. The cross-correlation between each pair of electrodes was calculated as follows:
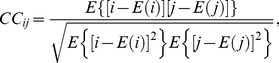
(1)where *i* and *j* are the time-series of two given electrodes having means 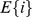 and 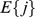 respectively. This calculation yields a 64×64 matrix of cross-correlations that are independent of firing rates [15].

Two electrodes were considered functionally connected if their pairwise cross-correlation exceeded a threshold that was calculated using a permutation test defined as follows. In a first step, the timings of extracellular spikes were randomly rearranged for each neuron independently across its entire recording time. For each neuron, spike trains were converted to a binary vector (“1” if a spike was emitted, “0” otherwise). This vector was then shuffled randomly by taking each spike and the inter-spike interval immediately following it, and moving it to a different location on the vector. This method of shuffling preserves the first-order statistics of both spike rates and interspike intervals. In a second step, the cross-correlations of shuffled spikes were computed to produce an estimate of chance correlations. Extracellular spikes were shuffled 100 times independently for every recording.

The greatest cross-correlation value found across all 100 runs of the permutation test was employed as threshold, above which a pair of electrodes was said to be functionally connected. Functional connectivity obtained by the permutation test was represented as an *N*×*N* adjacency matrix where *N* is the number of recording electrodes (Fig. 1D). In this matrix, diagonal entries (i.e., self-connections) were set to zero. Non-diagonal entries were set to either 1 (indicating above-threshold cross-correlation) or zero. An illustration of binary functional connections on a multi-electrode array is shown in Fig. 1E; only edges remaining after the permutation analysis are shown.

### Graph Analysis

**Figure 2:**
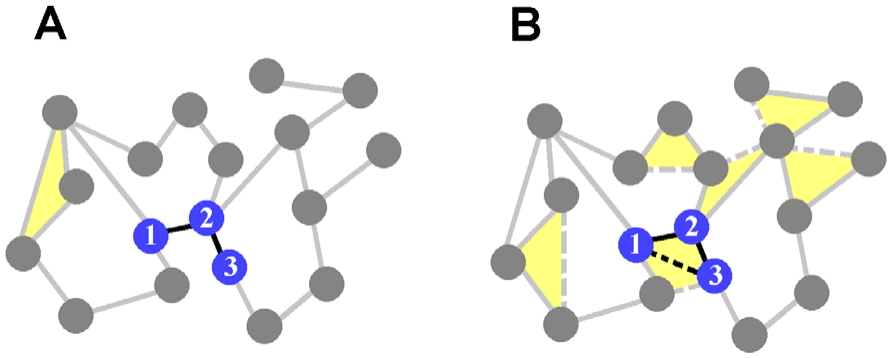
Adding connections between neighbouring nodes in a network. A. In this network, a majority of connections link nodes that are close together in space. **B.** New connections are added (dashed lines), resulting in a network with higher density, higher clustering, and shorter path lengths. The addition of connections between adjacent nodes thus alters the graph-theoretic properties of the network. A–B: yellow areas highlight triplets of nodes forming closed triangles, showing neighbours of a node that are neighbours of each other (clustering coefficient).

The adjacency matrix (obtained by the above-described permutation test) served as input to graph-theoretic analyses. In these analyses, we consider four different measures, namely the degree of each node, its clustering coefficient, its average path length, and the overall density of the network. The *degree* of a node refers to the total number of connections between that node and others in the network. For instance, node 1 in Fig. 2A has a degree of 3 because it connects to a total of 3 other nodes. *Path length* refers to the smallest number of intermediate nodes connecting a given pair of nodes in the network. For instance, the shortest path length linking nodes 1 and 3 in Fig. 2A is 1 because there is a single node separating them (node 2). Adding a direct link between nodes 1 and 3 (Fig. 2B, dashed black line) reduces the path length to zero. *Clustering coefficient* refers to the number of connections between neighbours of a node over the total possible number of connections. Here, the term ‘neighbours’ refers to pairs of nodes that are directly connected to each other (or in other words, have a path length of zero). For instance, node 2 (Fig. 2A) has a total of 4 neighbours, with a possible maximum of 6 connections between them (excluding bidirectional and self-connections). Because none of these neighbours are connected to each other, the clustering coefficient of node 2 is zero. Adding a link between nodes 1 and 3 (both neighbours of node 2) increases clustering to 1/6 = 0.17 (Fig. 2B). Formally, the clustering coefficient *C_i_* of a given node *i* is computed by dividing the number of connections between its neighbours, *n_i_*, over the total number of possible connections, *k_i_*(*k_i_*−1)/2, where *k_i_* is the degree of node *i*. Thus, for each node, the clustering coefficient is mathematically expressed as:
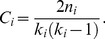
(2)


The average clustering coefficient over all nodes *i* is denoted *C*. Finally, we computed a measure of *density*, defined as the percentage of edges in a given functional network given all possible edges that could be present. Here, we define a *sparse* network as a network with less than 20% of all possible links (the justification for this criterion will become apparent in computational simulations below). For instance, the network of Fig. 2A has 18 nodes and therefore a capacity to accommodate up to 153 links. Given that this network has only 21 links, and hence a density of 21/153×100 = 13.73%, it is considered sparse.

For the purposes of the current work, we draw a distinction between two types of networks, namely random and small-world [16]. In a *random network*, all nodes have roughly the same degree, resulting in an overall binomial degree distribution. In *small-world networks*, the average clustering coefficient across nodes is markedly higher than in a random network with an equivalent distribution of degrees; the average path length, on the other hand, is comparable.

Networks may be designed such that the probability of a connection between two given nodes is highest when nodes are nearby in space, and lowest when nodes are distant. Networks wired in this way are termed *spatially-embedded* to reflect the influence of spatial distances on connectivity [17], [18]. For instance, the network of Fig. 2A is connected such that a majority of connections link together nearby nodes. In recordings of cortical networks, spatial distances reflect the physical proximity of electrodes on the multi-electrode array. Adding local connections in this network (Fig. 2B, dashed lines) yields a network with higher density, as evidenced by the increased number of links. The resulting network also shows higher clustering, as shown by the increased number of “closed triangles” between triplets of nodes (yellow shading) which highlight neighbours of a node that are also neighbours of each other. Finally, the resulting network has shorter path lengths, given the fewer number of intermediate links between nodes (e.g., the dashed line between nodes 1 and 3 in Fig. 2B reduced the path length between those two nodes). These effects will be examined in the Results section below.

Statistical testing was performed using the Wilcoxon rank-sum test with adjusted Bonferroni correction for multiple planned comparisons. This analysis yields a family-wise Type I error rate of 0.15 [19] and results in a critical *p* value of 0.0375. This test is employed to compare spike rates and network measures (density, clustering, and path length) across experimental conditions, and was chosen over parametric testing because distributions of spike rate violated the assumption of normality. In order to apply this test, we pooled together data from arrays within the same experimental condition (DIVs 14, 16, 17, and 21). The use of pooling is justified by the limited amount of variation across arrays (e.g., spike rates, Fig. 1C). The phi coefficient – a Pearson’s *r* estimate for binary data – was employed to examine the degree to which a given network’s pairwise connections correlated between experimental conditions [20]. The phi coefficient can be interpreted as a measure of pairwise wiring stability, calculated as:
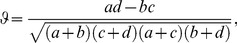
(3)where *a* is the number of pairs of electrodes that remained connected, *b* are the pairs that gained connections, *c* are the pairs that lost connections and *d* are the pairs that remained unconnected between two recordings. We computed Eq.3 for each culture, and obtained values of *a*, *b*, *c*, *d* by comparing DIV 14 to each later DIV (16, 17, and 21). This analysis yielded a separate value of phi coefficient and a test of statistical significance for each culture and each of DIVs 16, 17 and 21. Statistical significance was assessed using a chi-square test [21]:
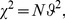
(4)where N = a+b+c+d and degrees of freedom are n-1 (given n number of observations). We corrected for multiple comparisons using a Dunn-Bonferroni correction that accounted for the number of cultures and the three later DIVs under consideration. In Results below, we report the mean value of φ 2 across cultures (denoted 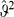), indicative of the proportion of shared variance between DIV 14 and a later time point. We also report the maximum value of phi coefficient across cultures (denoted φ max). All graph measures were computed using the Brain Connectivity Toolbox [22]. Additional custom software in the Matlab language served as an interface between experimental data and the toolbox.

## Results

### Reversible Effects of Preconditioning on Spike Rate

**Figure 3:**
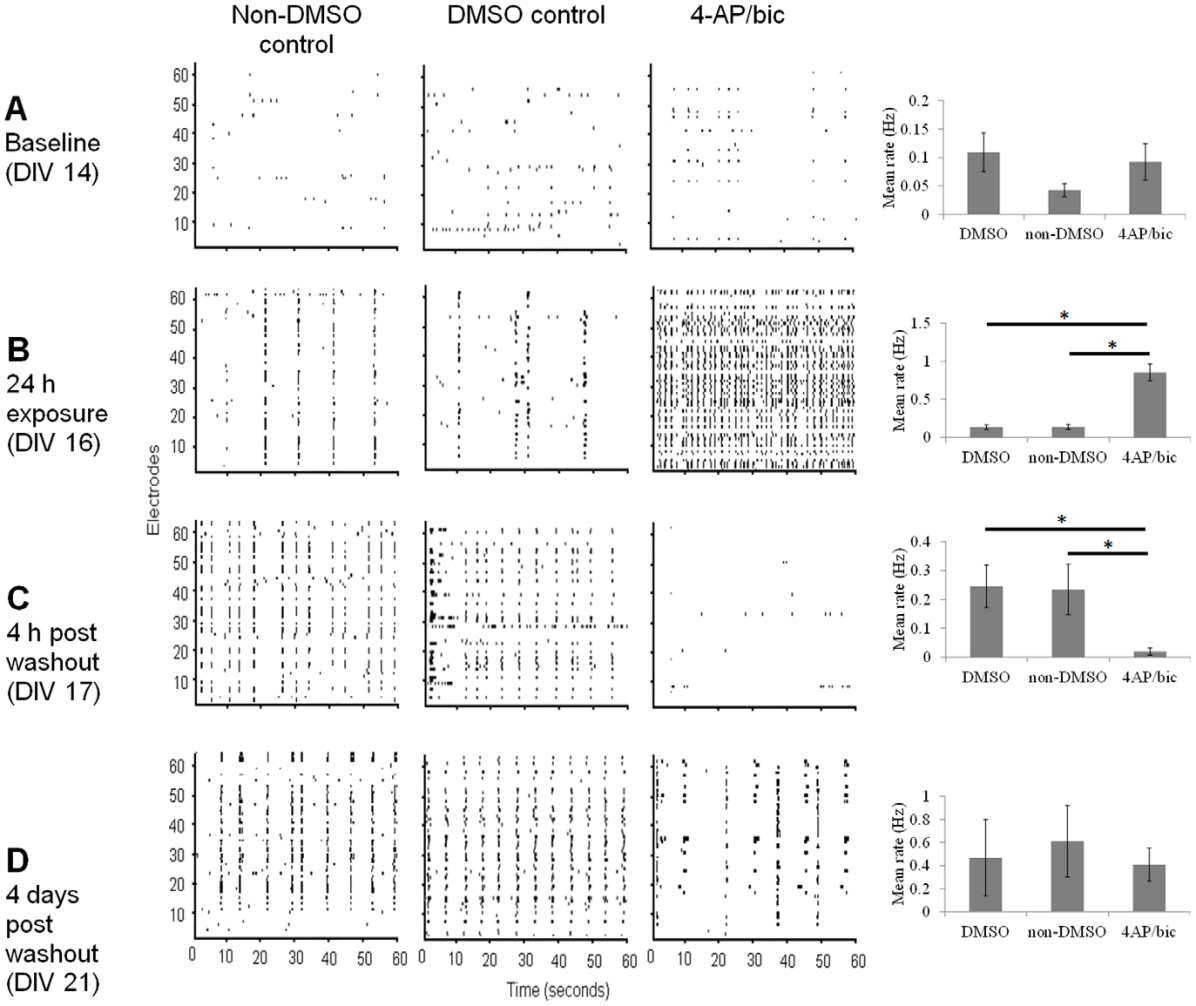
Representative rasters showing the timing of extracellular spikes across control conditions (DMSO and non-DMSO) and with 4-AP/bic exposure. In control cultures, activity begins to synchronize at DIV 14 (A); synchronization is maintained for the duration of the experiment (DIV 21). Application of 4-AP/bic induces hypersynchronization (B). Drug washout leads to a downregulation in activity (C), followed by recovery (D). Rightmost column are mean firing rates (* = *p*<.05). Vertical bars: SEM.

At DIV 14 (prior to experimental manipulations), mean spike rates per channel were typically low (<1 Hz) and followed a similar distribution across cultures (see Fig. 1C for a distribution taken from each DMSO recording at DIV 14, N = 5). Cultures exhibited brief periods of weakly synchronized activity interspersed by periods of relative quiescence (Fig. 3A). Due to violations in the assumptions of normality, the Wilcoxon rank-sum test for nonparametric data was used. Mean spike rates of the treated and control cultures were comparable at baseline (DMSO control vs. treated cultures, *p*>.21; non-DMSO control vs. treated cultures, *p*>.42). Recordings obtained 24 h after exposure to 4-AP/bic (DIV 16) show markedly increased synchronization compared to both DMSO and non-DMSO controls, accompanied by a surge in firing rates (Fig. 3B) (DMSO control vs. treatment, *p*<.008; non-DMSO control vs. treatment, *p*<.008). At DIV 17 (4 h post washout of 4-AP/bic), rates of activity for the treated cultures dropped below both DMSO and non-DMSO controls (Fig. 3C) (DMSO control vs. treatment, *p*<.02; non-DMSO control vs. treatment, *p*<.008). Finally, at DIV 21 (4 days post-washout of 4-AP/bic), the activity rates of treated cultures returned to levels comparable to both DMSO and non-DMSO controls (DMSO control vs. treatment, *p*>.69; non-DMSO control vs. treatment, *p*>.84) (Fig. 3D).

In summary, consistent with previous work, application of 4-AP/bic led to reversible effects on network activity rates [3]. In the next three sections, we consider whether graph-theoretic measures of functional connectivity, known to play a role in network communication, are also affected in a reversible fashion.

### Pairwise Cross-correlations Depend on Physical Distance and Follow a Lognormal Distribution

**Figure 4:**
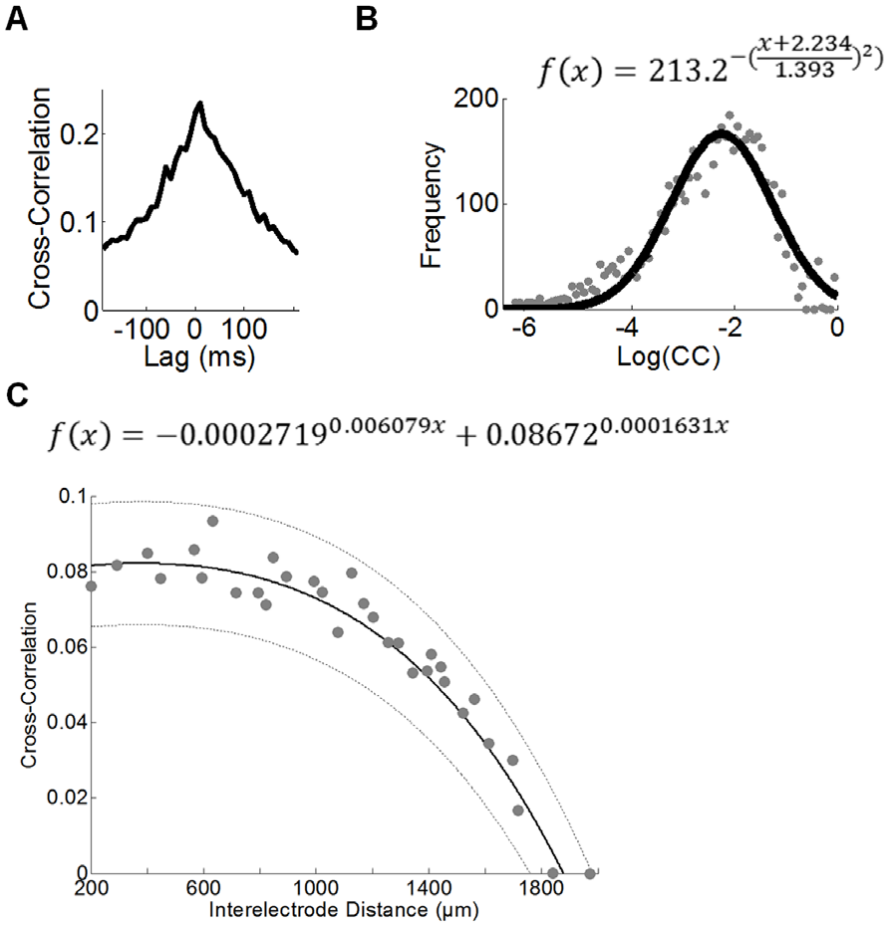
Baseline properties of functional connectivity in cortical networks. **A.** Representative cross-correlogram in a DMSO culture at baseline (DIV 14). The mean cross-correlation across all pairs of recording electrodes is highest at 0 ms time-lag. **B.** The distribution of pairwise cross-correlations (gray dots) is fitted by a lognormal distribution (solid line). Adjusted *r* ^2^ = .93. **C.** Relationship between cross-correlation and physical distances between electrodes on the MEA. Grey dots: individual data points of correlation between pairs of electrodes. Solid line: fitted exponential function. Dotted lines: 99% confidence intervals. Adjusted *r* ^2^ = .85. B–C are taken over all DMSO cultures from DIV 14 (N = 5).

In control (DMSO) cultures, cross-correlations between the timeseries of activity at pairs of electrodes were typically low and exhibited a similar distribution across cultures (see Fig. 1C for a distribution taken from each DMSO recording at DIV 14, N = 5). The mean spike cross-correlogram indicates a clear peak at a time lag of 0 ms (Fig. 4A) (similar results were obtained for non-DMSO cultures). Based on this result, we chose to estimate functional connectivity based on cross-correlations with 0 ms time lag; slight changes to this time lag did not drastically alter the resulting functional networks. The distribution of cross-correlations taken over all pairs of electrodes is well-fitted by a lognormal distribution (Fig. 4B). Unlike a Gaussian distribution, the lognormal distribution is asymmetric and decays much slower for large values [23]. While there were minor alterations in the parametric fit of the lognormal distribution across DIVs of the control cultures, a similar overall profile was observed.

Cross-correlations decreased exponentially as a function of distance between pairs of electrodes (Fig. 4C). This result provides supportive evidence for a spatially-embedded model of functional connectivity where the probability of a functional connection is dependent upon the physical distance between nodes (where a node represents an electrode on the multi-electrode array). We elaborate on such a model below. Distance-dependent correlations may reflect a decrease in the probability of synaptic connections as a function of physical distance between populations of neurons in the vicinity of each electrode [24]. Next, we consider how cross-correlations may be altered during and following 4-AP/bic treatment.

### Global Network Configuration Remains Stable Following Application of 4-AP/bic

Using the permutation test described in Materials and Methods, we converted pairwise cross-correlations to a binary network of connections between electrodes. Then, using the phi (*φ*) coefficient, we examined whether application of 4-AP/bic resulted in alterations (addition or deletion) of binary edges. In the DMSO control cultures, baseline functional connectivity (at DIV 14) was robustly correlated with later time-points at DIV 16 (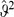 = .32, *φ*_ max_ = .72), DIV 17 (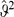 = .27, *φ*_ max_ = .63), and DIV 21 (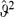 = .13, *φ*_ max_ = .55) (all three phi coefficients were statistically significant at *p*<.04 after Dunn-Bonferroni correction). A similar result was obtained for non-DMSO control cultures, where functional connectivity obtained at DIV 14 was correlated with that obtained at DIV 16 (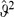 = .35, *φ*_ max_ = .79), DIV 17 (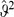 = .20; *φ*_ max_ = .58) and DIV 21 (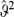 = .14, *φ*_ max_ = .61) (all three were statistically significant, *p*<.01). These results indicate that pairs of electrodes that are connected in earlier recordings tend to remain connected in later recordings, thus preserving stable functional connectivity over the course of *in vitro* development.

In cultures treated with 4-AP/bic, baseline functional connectivity (DIV 14) was correlated with connectivity obtained at 24 h after exposure (DIV 16; 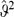 = .2, *p*<.01; *φ*_ max_ = .77) but was no longer correlated with connectivity obtained at 4 h washout (DIV 17; 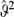 = .002, *p*>.42; *φ*_ max_ = .45), in part because of depressed levels of activity (Fig. 3C). Baseline functional connectivity was weakly (but significantly) correlated with connectivity at 4 days washout (DIV 21; 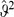 = .04, *p*<.01; *φ*_ max_ = .59), suggesting that effects of 4-AP/bic on functional connectivity were partly reversible.

**Figure 5:**
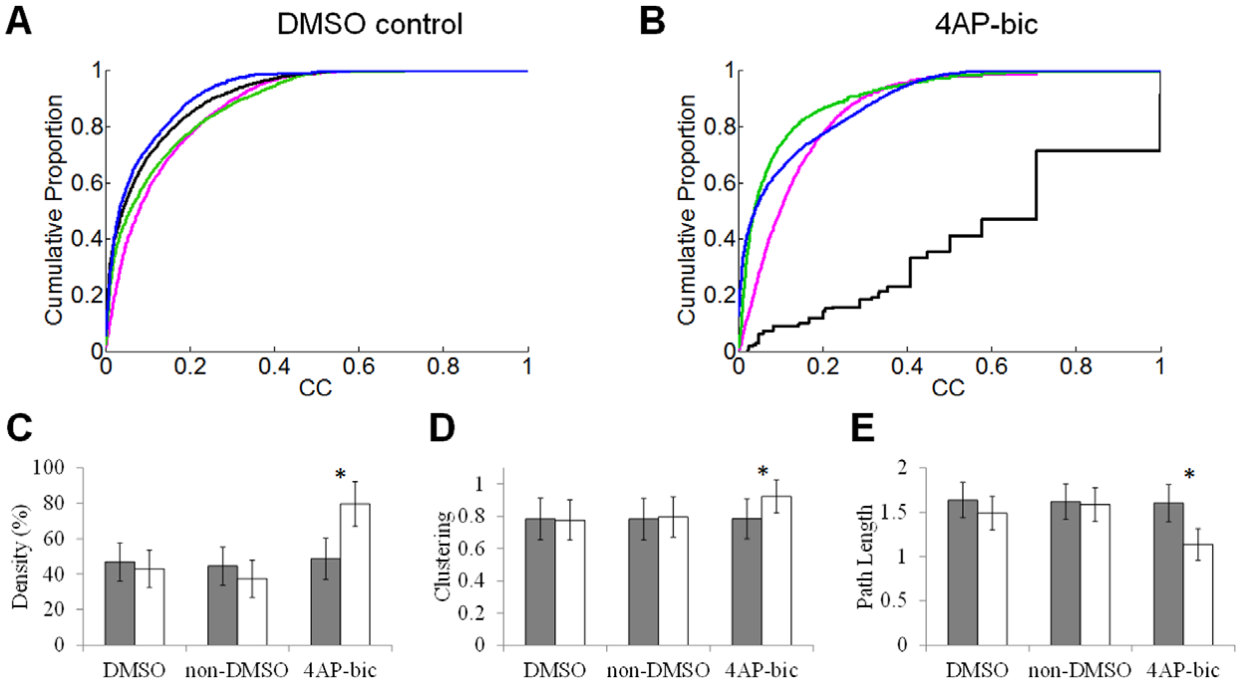
Alterations in functional networks exposed to 4-AP/bic. **A–B:** Cumulative distribution of cross-correlations for DMSO controls and 4-AP/bic networks in each recording session. Pink: prior to drug exposure (DIV 14); Green: 24 h after exposure (DIV 16); Black: 4 h washout (DIV 17); Blue: 4 days post washout (DIV 21). With the exception of the recording obtained 4 hours following washout of 4-AP/bic, all networks show a comparable distribution of cross-correlations. **C–E:** Alterations in network density (C), clustering (D), and path length (E) preceding exposure to 4-AP/bic (gray bars) vs. 4 days post washout (white bars). Vertical bars = SEM. * = *p*<.01 after Dunn-Bonferroni correction (Wilcoxon rank-sum test).

The transient nature of drug-induced changes in functional connectivity is supported by examination of the overall distribution of cross-correlations over time. In DMSO controls, the distribution of cross-correlations follows a lognormal distribution (see above); a similar distribution is observed at all time-points of recording (Fig. 5A). In preconditioned cultures, a marked departure from a lognormal distribution is observed at 4 h post washout (Fig. 5B, solid black line), largely due to depressed levels of activity following removal of 4-AP/bic (see Fig. 3C). This effect is transient, and the deviation from a lognormal distribution observed at 4 h post washout is no longer present 4 days post washout (DIV 21).

Taken together, the above results indicate that 4-AP/bic alters functional connectivity, but that these drug-induced changes are transient. The above analyses did not, however, consider more subtle aspects of functional networks that may be altered under 4-AP/bic. As illustrated by previous work [16], only a small number of rewired connections are required to transform a structured network into a small-world network, and this transition is undetectable at the local level. We address this issue in the next section by considering whether global measures of path length and clustering coefficient, associated with small-world networks, are altered as a result of preconditioning treatment with 4-AP/bic.

### Alterations in Small-world Properties of Functional Connectivity by 4-AP/bic

First, we aimed to determine whether baseline functional networks in the preconditioning group could be described as having small-world connectivity. We began by calculating the average path length (*L_real_* = 1.81) and clustering coefficient (*C_real_* = .67) of functional networks obtained by the permutation test (see Materials and Methods). These averages are taken over all DMSO cultures of DIV 14 (N = 5). Then, we randomized the connectivity of functional networks while maintaining intact the overall degree sequence [25] using Matlab code from the Brain Connectivity Toolbox [22]. We obtained a total of 100 randomized networks and, for each, calculated path length and clustering coefficient. When compared with these randomized networks, baseline functional networks of the preconditioning group were similar in terms of their average path length (*L_random_* = 1.71, *z*(293) = 1.903, *p*>.06) but had markedly higher clustering coefficients (*C_random_* = .39, *z*(293) = 11.167, *p*<5.9085E-29). These results are consistent with a small-world effect [9], [10]; comparable results were found for DMSO and non-DMSO cultures.

Next, we compared functional networks of the preconditioning group after exposure to 4-AP/bic with those of control DMSO cultures recorded at the same time-point (DIV 14). Both groups had statistically comparable values of average path length and clustering coefficient (Table 1 top row, Wilcoxon rank-sum test). Following washout of the drug (4 days post washout, DIV 21), the clustering of preconditioned cultures increased while their path length decreased compared to control cultures (Table 1, bottom row), showing a lasting effect of 4-AP/bic on the small-world organization of functional networks. This effect was accompanied by an increase in the density of functional connections compared to control cultures. Density was statistically higher in preconditioned cultures compared to controls at 4 days post washout.

**Table 1:** Alterations in graph measures with 4-AP/bic relative to DMSO controls at the same DIV.

	Clustering Coefficient	Path Length	Density
4h wash-in (DIV 14)	=	=	=
4 day washout (DIV 21)	UP	DOWN	UP

Note. Table shows the direction of statistically reliable differences between drug-treated cultures vs. DMSO controls (Wilcoxon rank-sum test, statistical criterion of *p*<.01 after Dunn-Bonferroni correction). Equal signs “ = ” indicate no statistically reliable differences.

While the above results describe alterations in preconditioned cultures compared to controls, we also examined within-group alterations between functional connectivity at baseline and 4 days post washout in preconditioned cultures. Results mirror those obtained above: functional networks of preconditioned cultures at 4 days post washout had higher density, higher clustering, and lower path length than networks of the same cultures taken at baseline (Fig. 5C–E). In control cultures (DMSO and non-DMSO), no statistically reliable changes were found between measures of functional networks taken at baseline versus 4 days post washout.

Taken together, results suggest that cultures treated with 4-AP/bic sustain lasting alterations in small-world functional connectivity. In order to account for changes in functional connectivity that impact path length, clustering, and density, the next section describes a spatially-embedded model where functional connections are added at random with a probability that is dependent on the physical distance between nodes. We show with this model that the above results suggest an increased randomization in the functional connectivity of 4-AP/bic networks compared to control networks.

### Spatially-embedded Model of Network Connectivity

We devised a simplified model of functional connectivity to account for alterations in small-world properties as a result of 4-AP/bic. The goal of this model is not to capture exact empirical values but rather to provide a framework for capturing changes in small-world functional connectivity as a network undergoes changes in density.

**Figure 6:**
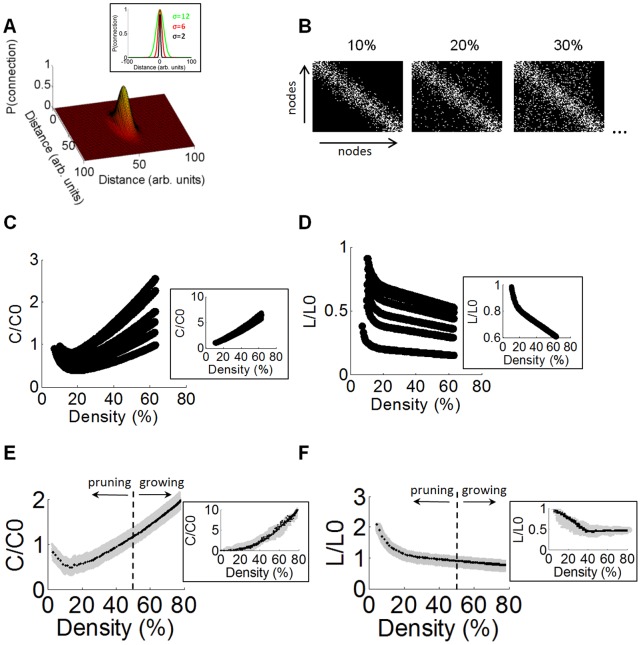
Spatially-embedded model of functional connectivity reproducing 4-AP/bic-induced alterations. **A.** Gaussian probability density function of the relationship between physical distance (arbitrary units) and the probability of two nodes being connected in the model. Inset: three different Gaussian functions are shown, with different values of standard deviation (σ). **B.** Connectivity maps for three different versions of the model where the density of connections is either 10%, 20%, or 30% of the total possible number of connections. The model was composed of 100 nodes. Binary ([0,1]) connections between nodes are shown in white. All three examples were generated with σ = 6. **C–D**: Using a spatially-embedded model of connectivity (see Main Text), we computed mean values of clustering coefficient (C) and path length (D) obtained with different percentages of connection densities ranging from a sparse model (10% density) up to a densely connected model (70% density). Different lines show models with values of σ = 12, 10, 8, 6, 4, 2 (from top to bottom) controlling the standard deviation of spatial embedding. Measures of clustering and path length were normalized by random networks that preserved degree sequence [25]. Note the U-shaped relation between clustering and density, whereas path length decreases monotonically as density increases. Inset: same as C-D, but obtained from a model with no spatial embedding. **E–F:** DMSO networks at baseline (DIV 14) exhibit a similar U-shaped relation between clustering coefficient and density (E) as the spatially-embedded model (σ = 2), while path length (F) decreases monotonically with density. Solid black lines: mean clustering coefficient and path length taken over DMSO baseline networks. Vertical dashed line: Mean density of original DMSO networks (without growing or pruning). Shaded areas: SEM. Inset = clustering and path length in analyses with no spatial embedding of the growing and pruning processes.

The starting point for this model is a set of *N* = 100 disconnected nodes (where no two nodes are connected to each other; simulations with *N* = 64 and *N* = 128 did not lead to qualitatively different results). These simulated nodes were positioned on a two-dimensional plane, thus accounting for spatial distances between nodes. We then gradually added connections between pairs of nodes, one link at a time. Nodes that were near each other had a greater probability of establishing new edges than nodes farther from each other. This was implemented in two steps. First, we drew a candidate connection between pairs of nodes selected with replacement from a uniform random distribution. Then, we computed the probability of maintaining that candidate connection in place by using a 2D Gaussian probability distribution that favoured the establishment of connections over short spatial distances (Fig. 6A). The above steps were repeated until a target density of connections was reached. This density was initially set to 10%, meaning that 10% of all possible pairwise connections were present (Fig. 6B, left panel). The target density was then gradually increased until it reached 80%, and at each increment of 1% we computed clustering and path length over the entire network.

In different simulations, we altered the width (σ) of the 2D Gaussian distribution relating the probability of connections to spatial distances. To draw a parallel with experiments, we consider that inter-electrode distances on the array are 200 µm (Fig. 1A) and that mean cross-correlations approach zero as inter-electrode distances increase beyond ∼1800 µm (Fig. 4C). By comparison, in the model, the probability of establishing a connection given a Gaussian function with σ = 2 approaches zero after ∼15 arbitrary distance units, corresponding to roughly the same ratio as experiments. All connections were stored in an *N*x*N* matrix of binary values (where “1” and “0” denote the presence and absence of a connection, respectively).

The relationship between clustering and density follows a U-shaped function (Fig. 6C). Clustering decreases as density increases from 10–15%, then gradually increases as density increases from 15–80%. Path length, on the other hand, decreases monotonically as density increases (Fig. 6D). The relationship between density and small-world properties provides a simple mechanistic explanation for the effect of 4-AP/bic on clustering and path length. In drug-treated cultures, network density increases from 48.75% at baseline to 79.59% at 4 days post-washout (DIV 21) (Fig. 5C). This increase is accompanied by an increase in clustering (Fig. 5D) and a decrease in path length (Fig. 5E). The model captures all these results in a unified fashion. In the model, an increase in density from ∼50% to ∼80% (values comparable to baseline and 4 days post washout cultures, respectively) leads to an increase in clustering (Fig. 6C) accompanied by a decrease in path length (Fig. 6D), in line with experimental findings. These results are straightforward to explain: a higher density of connections increases the incidence of shared connections amongst neighbours (leading to increased clustering) and decreases the number of intermediate steps to connect any pair of connected nodes in the network (leading to decreased path length). Similar results relating density, clustering and path length were found across various spatial-embedding functions (Fig. 6C–D).

In addition to capturing experimental findings, the model provides a testable prediction about network properties at low densities of functional connectivity. The model proposes that an *increase* from 10% to 20% density is accompanied by a *decrease* in clustering and a *decrease* in path length (Fig. 6C–D). This prediction is linked to the spatial embedding of connections in the model; an identical model without spatial embedding (i.e., where the spatial distance between neurons does not affect their probability of establishing a connection) does not yield a U-shape function relating density and clustering (Fig. 6C–D, insets).

We examined whether a U-shaped relation between density and clustering emerged from our experimental data. In a first series of analyses, we began with a DMSO functional network at DIV 14 (∼50% density, Fig. 5C). We then gradually added connections between pairs of electrodes following a distance-dependent probability (Fig. 6A, σ = 2). Each time a connection was added, we computed the small-world properties of the network (clustering coefficient and path length). A second series of analyses was designed to examine the relation between low connection densities and small-world properties. In these analyses, we began with a DMSO functional network at DIV 14 and gradually pruned connections, again following a distance-dependent probability with σ = 2. The growing and pruning analyses were repeated with all DMSO functional networks at DIV 14 (N = 5), and average clustering coefficients and path lengths were computed. The overall relation between density and clustering followed the characteristic U-shaped function found in computational simulations (Fig. 6E). We compared this relation with analyses that added and deleted connections but did not follow a distance-dependent probability. In this case, clustering increased monotonically with density (Fig. 6E, inset), suggesting that the characteristic U-shaped relation between clustering and density relies on distance-dependent connectivity. Path length did not yield a U-shape relation with density; this result was found both with and without distance-dependent connectivity (Fig. 6F). Overall, analyses of cortical functional networks corroborate the spatially-embedded model, and suggest that a U-shaped relation between density and clustering may be a property of a broad class of networks where connection probabilities depend on spatial distances amongst nodes.

## Discussion

We investigated the effect of a preconditioning drug treatment (4-AP/bic) on cortical neurons by combining multi-site recordings with analyses based on graph-theoretic measures of functional connectivity. Our results show alterations in the small-world properties of cultured neurons and represent, to our knowledge, the first demonstration of functional network organization under a neuroprotective preconditioning paradigm. Our results highlight the lasting consequences of 4-AP/bic on three measures of functional connectivity (path length, clustering coefficient, and density), despite the fact that rates of activity at individual electrodes return to baseline levels within 4 days post treatment.

Drug-induced alterations in path length, clustering, and density were captured by a simplified model of connectivity with random wiring and spatial embedding. In this model, density was gradually increased, with nodes that were near each other having a greater probability of establishing new edges than nodes that were farther apart. Consistent with multi-electrode array data obtained from preconditioned cultures, an increase in density in the model was accompanied by an increase in clustering and a decrease in path length. The model suggests that the addition of new edges as a result of 4-AP/bic follows a random process influenced by spatial distance. The presence of spatial embedding is in keeping with the distance-dependent strength of pairwise cross-correlations consistently found in cultures (Fig. 4C).

Simulations of network growth in a spatially-embedded model led to the prediction of a U-shaped relation between connection density and clustering coefficient, such that increasing connections from an initially low density (0–10%) led to a decrease in clustering followed by an eventual increase as density rose beyond 20%. This U-shaped relation was specific to spatially-embedded simulations, and did not emerge from networks whose growth was not constrained by spatial distance. We tested the prediction of a U-shaped relation between density and clustering by randomly pruning and growing DMSO networks obtained at DIV 14. When pruning and growing was performed according to a distance-dependent function, analyses of cortical networks yielded the characteristic U-shaped function observed in simulations. The effect was not produced when pruning and growing was performed without a distance-dependent function.

A U-shaped relation between density and clustering leads to the non-trivial finding that adding connections to spatially-embedded networks that are sparse (i.e., where the initial density is less than 20%) may lower the clustering coefficient, resulting in a network where pairs of connected nodes are less likely to share common neighbours. This finding is contrasted with the more standard notion that, in non-spatial networks, adding connections increases the probability that pairs of nodes will share common neighbours, thus increasing the clustering coefficient of the network and decreasing its path length [26].

Changes in the density of functional connections in drug-treated networks cannot at present be mapped directly to potential changes in synaptic connectivity or synaptic efficacies. Patch-clamp experiments show that the probability of a physical connection as well as the strength of a connection both follow a distance-dependent function [27]. The distance-dependent nature of synaptic connections, efficacies, and functional interactions place limiting constraints on the type of functional and anatomical connectivity that a network can exhibit. Furthermore, as illustrated both in our current simulations and analyses of cortical networks, distance-dependent networks exhibit characteristic alterations in clustering coefficient and path length as a result of the addition or deletion of connections.

In recent multielectrode recordings performed in macaques, two factors were identified as playing a prominent role in estimations of the small-world properties of functional networks on multielectrode arrays [28]. The first of these factors was the distance-dependent nature of functional connections on the array, as shown in our work. Accordingly, a small-world effect can emerge from networks without the need for explicit rewiring (in contrast to the original account of small-world networks) [16]. Distance-dependent connectivity allows for the formation of densely connected neighbours at the proximal scale, resulting in high clustering coefficient. In addition, distance-dependent connectivity allows for the formation of a few long-range connections, leading to a low path length.

A second factor contributing to a small-world effect is a bias caused by the non-homogeneous sampling of neurons under multielectrode recordings. This factor, however, is unlikely to account for our findings of drug-induced changes in small-world properties, given that any sampling bias would affect functional networks under all conditions [28].

4-AP/bic likely achieves neuroprotection by activation of an endogenous stress response. On the one hand, 4-AP elevates the concentration of positively charged potassium ions inside the postsynaptic terminal, which in turn increases the duration of neuronal spike discharges. On the other hand, bicuculline attenuates inhibitory signals in the neural network, increasing the probability of propagating action potentials. One possibility is that 4-AP/bic induces homeostatic plasticity that regulates overall levels of activity. Consistent with this idea, chronic exposure to bicuculline results in overuse hyposensitivity by downward synaptic scaling [29]. Still unknown, however, are the long-term effects of synaptic scaling on the emergent organization of functional networks.

Our work opens the door to questions on the relationship between functional connectivity and neuroprotection provided by preconditioning. Are there certain patterns of functional connectivity that promote neuroprotection in the face of ischemic injury? And are these patterns of functional connectivity maintained after an insult? Addressing these important questions will require further work that combines neuroprotection, ischemic injury, and graph analysis.

The study of functional connectivity on multi-electrode arrays has been expanding steadily in recent years. In one study, authors proposed a model of neuronal injury based on elevated glutamate levels. Four days following glutamate exposure, functional networks underwent a transition from small-world to random organization, characterized by a drop in clustering coefficient and a Gaussian degree distribution [10]. However, this analysis was performed on cultures in which some neurons died, whereas the current study examined a neuroprotective phenotype. In other studies, neurons were electrically stimulated in order to induce plasticity, and functional networks were shown to undergo pathway-specific changes consistent with potentiation and depression [30], [31]. Despite these advances, many questions remain unanswered. Perhaps most importantly, we have a poor grasp of the relation between measures of functional connectivity and the underlying synaptic architecture of *in vitro* networks. Progress in this area will allow us to draw links between synaptic plasticity and its impact on functional connectivity at the scale of neuronal circuits.

The ubiquity of small-world properties in multiple spatial scales and algorithms for reconstructing functional connectivity suggests that these properties constitute an adaptive organization in brain circuits. There is, however, no consensus as to why they are so prevalent. One hypothesis is that small-world networks allow for rapid information processing because of their short path length [5], [24]. Another possibility is that these properties have no defined role, but simply represent the byproduct of competitive processes in development [32]. Regardless of their ultimate functional purpose, it is widely accepted that loss of small-world organization results in disruptive changes at both the neural [10] and cognitive [5] levels. Understanding the impact of drug interventions on functional networks is a crucial step towards the design of efficient strategies for maintaining intact neural processes that are central to cognitive and behavioral outcomes.
